# Applying Deep Learning Model to Predict Diagnosis Code of Medical Records

**DOI:** 10.3390/diagnostics13132297

**Published:** 2023-07-06

**Authors:** Jakir Hossain Bhuiyan Masud, Chen-Cheng Kuo, Chih-Yang Yeh, Hsuan-Chia Yang, Ming-Chin Lin

**Affiliations:** 1 Graduate Institute of Biomedical Informatics, College of Medical Science and Technology, Taipei Medical University, Taipei 11031, Taiwan; d610105005@tmu.edu.tw (J.H.B.M.); m610105009@tmu.edu.tw (C.-C.K.); d610104001@tmu.edu.tw (C.-Y.Y.); 2 International Center for Health Information Technology (ICHIT), College of Medical Science and Technology, Taipei Medical University, Taipei 11031, Taiwan; 3 Clinical Big Data Research Center, Taipei Medical University Hospital, Taipei 11031, Taiwan; 4 Department of Neurosurgery, Shuang Ho Hospital, Taipei Medical University, New Taipei City 23561, Taiwan; 5 Taipei Neuroscience Institute, Taipei Medical University, Taipei 11031, Taiwan

**Keywords:** clinical note, natural language processing, deep learning, convolutional neural network, diagnosis code

## Abstract

The International Classification of Diseases (ICD) code is a diagnostic classification standard that is frequently used as a referencing system in healthcare and insurance. However, it takes time and effort to find and use the right diagnosis code based on a patient’s medical records. In response, deep learning (DL) methods have been developed to assist physicians in the ICD coding process. Our findings propose a deep learning model that utilized clinical notes from medical records to predict ICD-10 codes. Our research used text-based medical data from the outpatient department (OPD) of a university hospital from January to December 2016. The dataset used clinical notes from five departments, and a total of 21,953 medical records were collected. Clinical notes consisted of a subjective component, objective component, assessment, plan (SOAP) notes, diagnosis code, and a drug list. The dataset was divided into two groups: 90% for training and 10% for test cases. We applied natural language processing (NLP) technique (word embedding, Word2Vector) to process the data. A deep learning-based convolutional neural network (CNN) model was created based on the information presented above. Three metrics (precision, recall, and F-score) were used to calculate the achievement of the deep learning CNN model. Clinically acceptable results were achieved through the deep learning model for five departments (precision: 0.53–0.96; recall: 0.85–0.99; and F-score: 0.65–0.98). With a precision of 0.95, a recall of 0.99, and an F-score of 0.98, the deep learning model performed the best in the department of cardiology. Our proposed CNN model significantly improved the prediction performance for an automated ICD-10 code prediction system based on prior clinical information. This CNN model could reduce the laborious task of manual coding and could assist physicians in making a better diagnosis.

## 1. Introduction

The World Health Organization (WHO) has published and maintains a list of diagnostic classifications called the International Classification of Diseases (ICD), which gives each illness a unique code [1,2]. ICD is a system of categorization and coding for disease. The ICD-10 version consists of more than 70,000 codes [3]. ICD codes have been broadly adopted by healthcare providers for healthcare reimbursement and retrieving diagnostic information [4,5]. Medical coders need to extract key information and assign correct codes based on categories within an electronic medical record (EMR) [6]. The complex structure and amount of information in EMRs significantly increase the difficulty of manual coding. In the outpatient departments of hospitals, physicians manually assign ICD codes based on the information given in clinical notes; however, this is challenging, time-consuming, and prone to error [7]. Billing issues and underpayments can result from coding errors [8].

Automatic coding systems have become more popular as a result of recent developments in the fields of deep learning (DL) and natural language processing (NLP) systems. DL has shown promising outcomes in text classification [9,10,11]. EMR is a great source of text data as it includes clinical notes and discharge notes. Clinical notes, which include SOAP (subjective component, objective component, assessment, and plan) notes, drug lists, and ICD-10 codes, are a crucial resource for determining the nature of health issues. In recent years, automatic ICD coding has become a significant area of clinical medicine study. As a result, there is a huge need for an automatic ICD coding system.

Recent research has focused on methods linked to deep learning. The deep learning model can produce astounding outcomes in computer vision [12]. Zhang et al. [13] revealed a gated recurrent unit (GRU) network to predict medication on the basis of the disease codes. Wang et al. [14] used natural language processing to classify ICD-10 cm codes from hospital text data and achieved an F1-score of 0.62. Chen et al. [15] proposed a deep neural network (DNN) model to predict the ICD-10 clinical modification code and achieved an F1-score of 0.715. Wang et al. [16] revealed word2vec to predict ICD-10 cm code and achieved an F-score of 0.67. 

The most popular deep learning architecture for explaining issues with natural language processing is the convolutional neural network (CNN) [17,18]. In order to categorize sentences using word2vec, Kim [19] presented a convolutional neural network model. A deep learning strategy was suggested by Gangavarapu et al. [20] to categorize the ICD-9 code in the nursing notes. A CNN model based on deep learning was utilized by Chen et al. [21] to predict the incidence of cerebral infarction from hospital data. The deep learning-based CNN techniques of Moons et al. [22] promoted the use of the discharge report for ICD-9 code classification. 

Nursing notes are gathered using a multi-label classification system to anticipate ICD-9 [20]. Nursing notes are used in numerous studies to predict ICD-9 codes [23]. An app was created by Mauch et al. [24] to predict incisional hernias. Discharge summaries were used by Kavuluru et al. [25] to predict the EMRs’ ICD codes. A deep learning algorithm was utilized by Li et al. [26] to forecast bone disease using EHR. Based on EHR data, Jin et al. [27] created a deep learning model to predict cardiac disease. 

A research paper deals with the NLP methods to predict medical specialties from the unstructured text notes of a university hospital [28]. Vinod, P., et al. demonstrated a deep learning model from clinical text data [29]. Teng, F., et al. developed a deep learning model to predict ICD codes from free text data [30].

In the traditional machine learning approach for text classification, various processes are employed for feature extraction, such as TF-IDF, one-hot encoding, counts, and so forth. Deep learning, on the other hand, serves as a universal tool for optimizing the selection of these features. In this study, we demonstrate that the application of deep learning can yield significant improvements in text classification. There are many synonyms, acronyms, and typos in EMRs. Based on our experience, the vocabulary used in each department or by each doctor is different. If using rule-based methods, it will need to create a set of “rules” for each doctor.

Automatic ICD-10 coding has been the subject of numerous investigations. The model achievement of these approaches to automatically predicting ICD-10 codes has limitations, though, and it is not clinically sensible. By developing disease prediction models, healthcare professionals can gain insights to develop treatment plans and enhance the overall quality of patient care. The lack of clinically satisfactory studies motivates us to conduct this research. This prediction can identify the appropriate diagnosis codes with a probability score from text data. Then, the physician can identify proper diagnosis codes efficiently. This model can help in the completeness (predict missing diagnosis from EHR) of diagnosis. We focused on a multi-label text categorization system for medical records in this work. The best ICD-10 codes were determined using a “top 10” ranking approach with a likelihood score for each prediction. Additionally, for each prediction, we manually checked the missing ICD-10 codes. 

The use of a convolutional neural network (CNN) based multi-label text classification, with data taken from SOAP notes and drug lists, to predict ICD-10 codes is a new approach in this field. The objective of this work was to build a deep learning model that can assist doctors in choosing the most pertinent ICD-10 codes.

## 2. Methods

**Figure 1:**
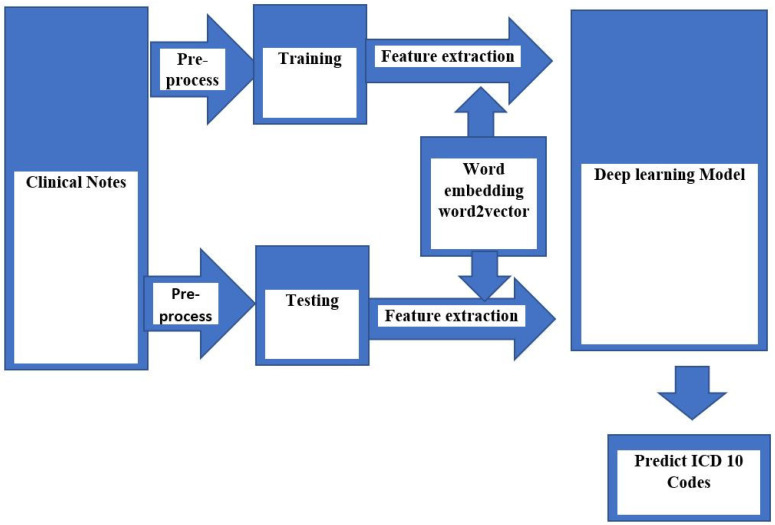
Study Design.

**Dataset:** This is a retrospective cohort study of patients. We collected clinical notes from the EMRs of a university hospital in Taipei, Taiwan. The dataset consists of clinical notes from the outpatient department (OPD) in the year 2016 (from January to December). The clinical notes consisted of SOAP (subjective component, objective component, assessment, and plan) notes, drug lists, and diagnosis lists (ICD-10 code). In the study, we focused on the three variables of SOAP notes, drug lists, and ICD-10 codes. The total number of clinical records was 21,953, which spanned five departments (Neurology, Psychiatry, Nephrology, Cardiology, and Metabolism). A total of 20,173 (90%) records were used for dataset training, while 1780 (10%) were used as a test set. In this study design (Figure 1), we selected 21,953 clinical notes from five departments to develop a deep learning-based CNN model. The Taipei Medical University Joint Institutional Review Board (TMU-JIRB) approved this study. 

**Data Pre-processing:** Clean and relevant information was desired for developing a deep learning model. Punctuation, supplemental spaces, infrequent words, stop words, and redundant components were all eliminated. The Natural Language Toolkit package (version 3.8) and the Python package (version 3.8) were used to perform pre-processing before tokenizing the text [31,32].

**Extraction of feature:** In this study, we extracted features from the raw data using the word2vec method [33], and then we trained deep learning models using those features. Word embedding is a form of word representation that maps words onto real-number vectors by representing words in a vector space with many dimensions. 

Models that create word embeddings are part of word2vec. Both the Continuous Bag of Words (CBOW) and the skip-gram designs are used by word2vec. While skip-gram attempts to predict several context words from a single input word, CBOW aims to predict a single word from a defined window size of context words.

**Figure 2:**
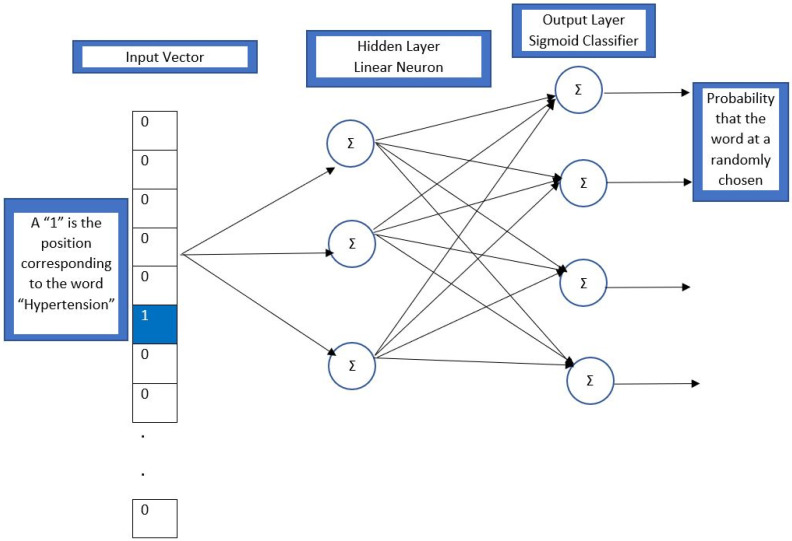
Architecture of neural network.

The skip-gram model transforms a one-hot vector for each word based on a corpus of text data. The one-hot vector is used to convert a word into a vector made up entirely of 0; one coordinate, which represents the string, is equal to 1. A neural network with a single hidden layer receives the one-hot vector input. To change a distributed representation of words and employ a vector with a various number of dimensions, a sequence of text is used. Then, each word is carried out at random with different weight distributions among the components. The vector size is equal to the number of distinct words in a text (Figure 2).

The input layer has a dimension of 1 × V, where V is the number of words in the corpus vocabulary (i.e., one-hot depiction of the word). The input layer is transformed into the hidden layer using the weight matrix. This hidden layer has a dimension of 1 × E, where E is the selected size of the word embedding. Finally, the weight matrix transforms the hidden layer into the output layer. In the hidden layer, we employed the sigmoid function. The last layer has a size of 1 × V, and each value in the vector represents the likelihood score of the target word in that position. We now have a weight matrix W of dimension V × E, following the training of the entire vocabulary. This matrix links the input layer to the hidden layer. The weight matrices are then adjusted as the model gains experience in predicting the main word.

**Figure 3:**
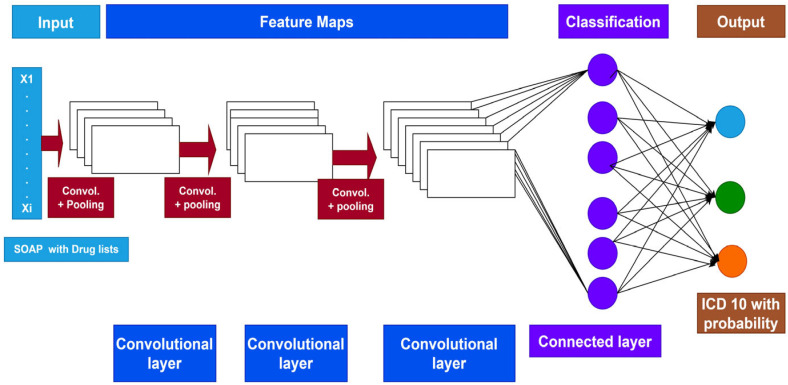
Deep learning CNN architecture.

**Development of Deep Learning Model:** In the current study, we predicted ICD-10 codes using a CNN classification model based on deep learning. First, word2vec was used to create dense, low-dimensional feature vectors from the input words. We set the parameter fixed by sample length = 200 Matrix length and the vector dimension created by word2vec to 128 in the training. To capture various n-gram characteristics, the filter window sizes in the convolutional layer were 1, 2, 3, 4 and 5 with a fixed filter window width of 128. The most important features were then retrieved from the feature map for classification in the maximum pooling layer (Figure 3).

**Figure 4:**
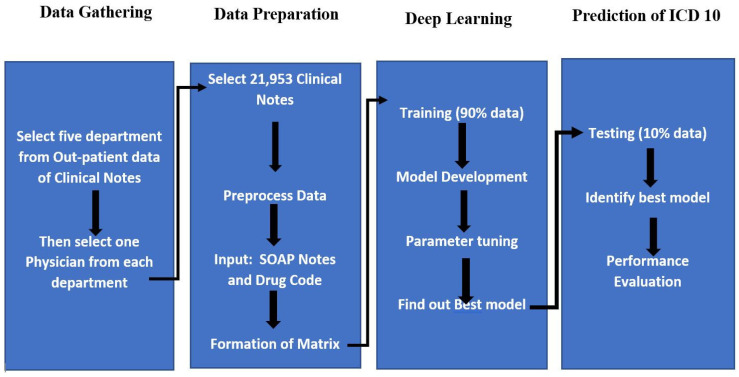
The overall layout of the study.

The SOAP notes and drug lists used as inputs and the ICD-10 codes as output were used in this study to test the assignment as a multi-label text classification issue. Python and Keras [34] were utilized to carry out the word embedding word2vec CNN work for the multi-label text classification challenge. The final layer of CNN that we used was the sigmoid activation function. ICD-10 codes were predicted using a ranking (top 10) approach. This ranking system is able to categorize the best diagnoses in chronological order. For the training set, 90% of the data were used, and for the test set, 10% was utilized. Figure 4 depicts the study’s overall design.

**Performance measure:** The deep learning-based CNN model performance was measured by three metrics (precision, recall, and an F-score).

***Precision*:** This denotes the proportion of actual positive results to all positive results. Equation (1):

(1) $$Precision=\frac{True\;positive}{True\;positive+False\;positive}$$

***Recall*:** The recall denotes the number of positive results made out of all positive results in the dataset. Equation (2):

(2) $$Recall=\frac{True\;positive}{True\;positive+False\;negative}$$

***F-score*:** The harmonic mean of recall and precision is an F-score. Equation (3):

(3) $$F-score=\frac{2\times Precision\times Recall}{Precision+Recall}$$

## 3. Results

**Characteristics of Data:** We retrospectively collected the data of all patients who visited a university hospital between January and December 2016. A total of 21,953 clinical notes were included in this study (Table 1). In our dataset, the majority of records (6027) were from the neurology department. Then, the psychiatry department provided 5789 records; the nephrology department provided 3707 records; the cardiology department provided 3668 records, and the metabolism department provided 2762 records in the total dataset. The age range of patients was between 10–101 years. 

**Performance of Model:** Precision, recall, and an F-score were used to gauge how well the deep learning-based CNN model performed. With a precision of 0.96, a recall of 0.99, and an F-score of 0.98, the Department of Cardiology performed the best, followed by the Departments of Metabolism (F-score of 0.86), Psychiatry (F-score of 0.75), and Neurology (F-score of 0.71) (Table 2). Batch 64 produced a good model performance with a precision of 0.66, a recall of 0.94, an F-score of 0.77, and an accuracy of 0.94 (Table 3). Accuracy in training and testing is 94% using 70/30 data (Table 4).

**Evaluation:**

We evaluated our deep learning-based CNN model to identify its overall accuracy and appropriateness; a manual review was also conducted. Our CNN model appropriately predicts the necessary ICD-10 codes. 

**Figure 5:**
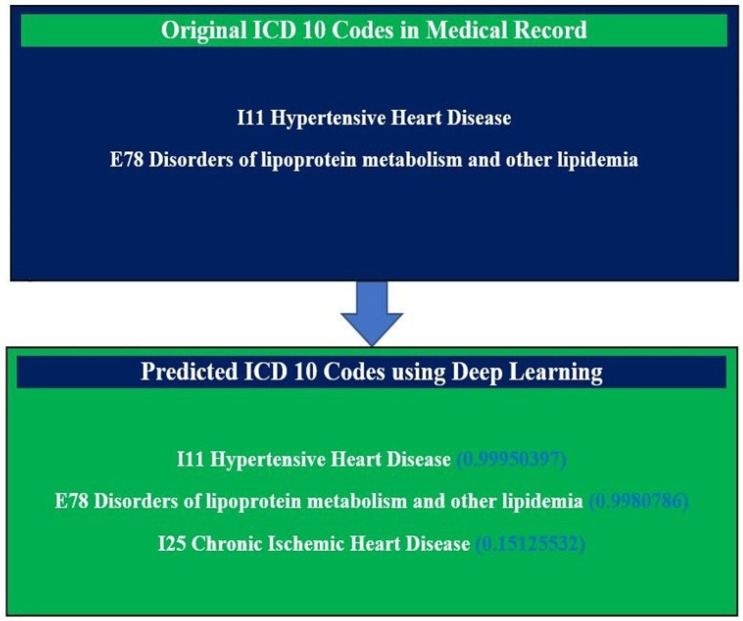
Evaluation of the performance of deep learning to predict ICD 10 codes with a probability score.

We further investigated the predicted ICD-10 codes and found that the CNN model has a high likelihood of accurate ICD-10 code prediction based on the words. For example, in the clinical notes for Figure 5, there were certain words used for chronic ischemic disease; our model was able to identify these words and correctly report a missing ICD-10 code. In the original medical records, the physician entered two diagnosis codes (ICD-10 codes), four drug codes, and SOAP notes for a patient. However, the CNN model predicted the appropriate disease codes (with one extra ICD-10 code) with a probability score based on clinical notes (Figure 5).

**Figure 6:**
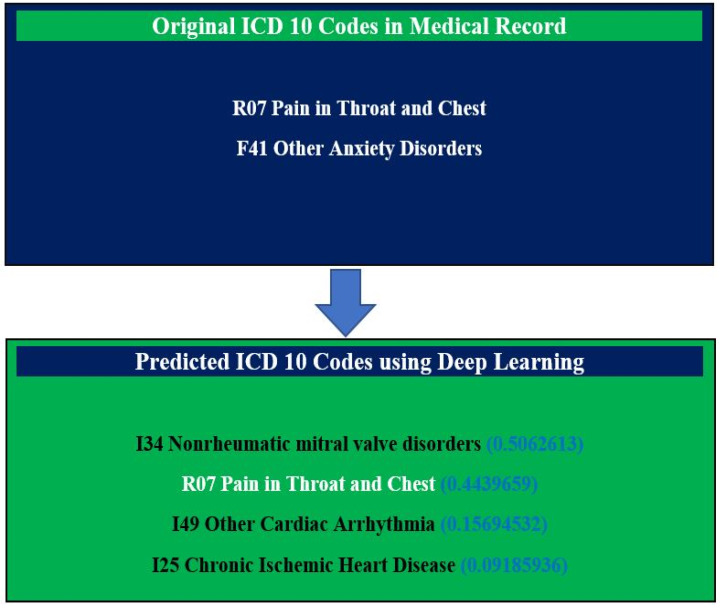
Evaluation of the performance of deep learning to predict ICD 10 codes with probability scores for missing diagnoses.

For the data in Figure 6, certain words were found in the clinical notes for cardiac arrythmia, nonrheumatic mitral valve disorder, and chronic ischemic heart disease; the CNN model has a high chance of accurate ICD-10 code prediction based on the words and drug history. In the original clinical notes, the physician input two ICD-10 codes, five drug codes, and a SOAP note for a patient. However, the CNN model predicted the appropriate disease codes (three extra ICD-10 codes) with probability scores based on clinical notes (Figure 6). Thus, our model predicted the missing ICD-10 codes that could help the physician in better decision making.

## 4. Discussion

**Main Findings:** Our study was designed to develop a deep learning-based CNN model to identify diagnosis codes automatically from clinical notes of medical records. This model achieved satisfactory performance in predicting ICD-10 codes using SOAP notes and drug lists. The performance of the deep learning model was the highest in the Department of Cardiology (with a precision of 0.95, a recall of 0.99, and an F-score of 0.98), followed by the Department of Metabolism (with a precision of 0.78, a recall of 0.97, and an F-score of 0.86), the Department of Psychiatry (with a precision of 0.64, a recall of 0.91, and an F-score of 0.75), and the Department of Neurology (with a precision of 0.60, a recall of 0.85, and an F-score of 0.71).

**Compared to Previous Research:**

A deep learning model was used in our study to forecast ICD-10 codes from clinical notes. By identifying relevant ICD-10 codes based on clinical notes, our CNN model has the enormous potential to improve the accuracy of ICD-10 coding and decrease the amount of human coding. The model performance in this study can correctly predict missing ICD-10 codes and is clinically satisfactory. Prior research has been conducted to evaluate the potential of deep learning-based models for ICD-10 code prediction. These deep learning models did not, however, perform in a clinically desirable manner.

A Micro F1 score of 63.42 was obtained for 50 distinct ICD-9 block codes in a study by Moons et al. [22] using a deep learning-based CNN model to predict ICD-9. The ICD-9 codes were categorized in their study using discharge summaries. A hierarchical deep learning model was created by Shi et al. [35] to predict ICD codes from discharge notes, and this model received an F-score of 0.53 for 50 ICD codes. 

Suo et al. [36] employed convolutional neural networks and their model had an accuracy of up to 0.74 in predicting diabetes mellitus, obesity, and chronic obstructive pulmonary disease. A convolutional neural network model was utilized by Cheng et al. [37] to forecast the future recurrence of chronic heart failure and chronic obstructive pulmonary disease. 

A light gradient-boosting machine (LightGBM) was employed by Diao et al. [38] to automate ICD-10 categorization from discharge summaries, and their best model produced a macro-averaged F1 (Macro-F1) score of 88.3%. Wang et al. [16] proposed a deep learning model to predict ICD-10 clinical modification codes from EHR data and their model achieved an F-score of 0.67.

A deep learning model was put forth by Rashidian et al. [39] to predict ICD-9 codes from EHR data. In this study, they predicted diabetes, acute renal failure, and chronic kidney disease using data from demographics, lab findings, and prescription information. Their deep learning model received an F1-score of 80.04 for diabetes, an F1-score of 66.86 for acute renal failure, and an F1-score of 75.77 for chronic kidney disease. 

A deep learning method to predict ICD-19 codes from discharge summaries was put forward by Li et al. [40]. On the MIMIC-II dataset, their model obtained a micro F-measure of 0.335, while on the MIMIC-III dataset, it obtained a micro F-measure of 0.408. A deep learning model was also put forward by Choi et al. [41] to predict diagnosis codes using EHR data. A recurrent neural network model was applied in their study, which had a 79% recall rate.

In their study of Word2Vec convolutional neural networks for ICD-9 coding prediction, Hsu et al. [42] reported a micro F1 score of 0.76 for 19 different ICD-9 chapter codes from the discharge summary. The accuracy for 19 different ICD-9 chapter codes was 0.833, according to Gangavarapu et al. [43], who utilized a deep learning model trained on nursing notes. In order to resolve the multi-class labeling and multi-label classification technique, Samonte et al. [44] used an enhanced hierarchical attention network (EnHAN) and the word embedding method. Their model had an accuracy of 0.841. 

A strategy to predict ICD-9 codes using clinical notes was put forward by Obeid et al. [45], and their model achieved an F-score of 0.769. From a subjective aspect of clinical notes, Hsu et al. [46] suggested a deep learning model and obtained an accuracy of 0.409 for 2017 different ICD-9 codes. For 2833 ICD-9 codes, Xie et al. [47] employed a deep learning model trained on the diagnosis description and attained a sensitivity of 0.29. From a subjective feature of clinical notes, Singaravelan et al. [48] developed a deep learning model and attained a recall of 0.57 for 1871 ICD-9 codes. 

Zeng et al. [49] developed a deep learning model to predict ICD-9 codes from discharge summaries and obtained an F1 score of 0.42 for 6984 ICD-9 codes. The application of deep learning to predict ICD-9 codes by Huang et al. [50] resulted in an F1 score of 0.6957 for 10 ICD-9 codes. In their investigation, ICD-9 codes were predicted using clinical notes. 

Our study uses 1131 ICD-10 codes in the process of ICD-10 prediction. In this study, we predicted ICD-10 codes using SOAP notes and drug lists. This comparison demonstrates the originality of our research. We reviewed (Table 5) the deep learning model performance of work on ICD code prediction. It shows that our work is superior to previous investigations. The table compares the results of our investigation to those of earlier studies.

**Strength of the study:** There are a few advantages of this study. The first thing to note is that this is the first study to evaluate the performance of a CNN model built on deep learning for ICD-10 code prediction utilizing SOAP notes and medication lists. Second, our CNN model can accurately identify ICD-10 codes based on SOAP notes and prescription lists, which can assist clinicians in correctly identifying ICD-10 codes. Thirdly, our model offers a list of the top 10 diagnoses with probability scores, with the assumption that a diagnosis with a higher probability score will be more accurate. Doctors can therefore choose a diagnosis from the diagnosis list.

**Limitation of the study:** There are obviously some drawbacks to this study. First, data from a single university hospital was employed in our model. Data from other hospitals might have an impact on the model’s performance because their clinical notes may have different writing styles and disease identification patterns. Second, the dataset we used came from five departments using medical records. The dataset is not particularly large. Third, we did not validate our research with data from an outside source. Lastly, we used only one method in our study.

**Future perspective:** Our deep learning-based CNN model behaves as an assistance tool that helps physicians in better decision making. This model could reduce the manual entry of ICD-10 codes. As our findings are promising, we will use more data to make the model more effective. In the future, we will focus on more advanced NLP methods such as BERT (bidirectional encoder representations from transformers) to predict ICD codes.

## 5. Conclusions

In this study, we built a CNN-based deep learning model to predict ICD-10 codes based on data from the SOAP notes and drug lists of clinical notes. The cardiology department’s prediction model performed best with a precision of 0.96, recall of 0.99, and F-score of 0.98. Clinically good performance is achieved by the prediction model. Textual data are often complex, of variable lengths, and with nuances in meaning. Our deep learning models can effectively handle such complexities by capturing contextual dependencies and long-range dependencies within the text. They can capture a text’s non-structural (SOAP) and structural information (drug list), enabling a more accurate classification. This model recommends utilizing disease lists chronologically to assist physicians in selecting appropriate ICD-10 codes. This CNN model, which is based on deep learning, performs better than models applied in earlier experiments. Our research established that drug lists and SOAP notes have a significant role in predicting ICD-10 codes. With the help of our deep learning model, we can classify diseases appropriately. A quick and accurate ICD-10 coding decision can be made by doctors with the help of this approach.

## Figures and Tables

**Table 1:** Characteristics of data.

Characteristics	Frequency (n)
Total number of clinical notes from Doctors of five Departments	21,953
Doctors’ notes from Neurology	6027
Doctors’ notes from Psychiatry	5789
Doctors’ notes from Nephrology	3707
Doctors’ notes from Cardiology	3668
Doctors’ notes from Metabolism	2762
Number of ICD 10 codes	1131
Number of Drugs	807

**Table 2:** CNN model performance for the doctor from five departments.

Name of Department	Test Cases	Number of ICD-10 Codes	Number of Drugs	Precision	Recall	F-Score	Accuracy
Doctors’ notes from Cardiology	284	148	145	0.96	0.99	0.98	0.99
Doctors’ notes from Metabolism	307	155	136	0.78	0.97	0.86	0.97
Doctors’ notes from Psychiatry	475	193	128	0.64	0.91	0.75	0.91
Doctors’ notes from Neurology	282	358	177	0.60	0.85	0.71	0.85
Doctors’ notes from Nephrology	432	277	221	0.52	0.88	0.65	0.88

**Table 3:** Model performance using different batch sizes for one doctor.

Batch	Accuracy	Precision	Recall	F-Score	Loss	Error Rate	Computational Time (Minutes)
64	0.94	0.66	0.94	0.77	0.03	0.06	14
128	0.89	0.45	0.89	0.60	0.05	0.11	14
512	0.88	0.44	0.88	0.59	0.06	0.12	14
1000	0.84	0.40	0.84	0.54	0.07	0.16	14
1024	0.84	0.39	0.84	0.53	0.07	0.16	14
2000	0.81	0.38	0.81	0.52	0.08	0.19	14
2048	0.81	0.36	0.81	0.50	0.08	0.19	14

**Table 4:** Training and testing accuracy of the one doctor model.

Training Accuracy	Testing Accuracy
0.94	0.94

**Table 5:** Comparative evaluation of different studies.

Work	Data	Method	Target Variable	Performance Measure
Hsu et al. [42]	Discharge summary	Deep learning	(i) 19 distinct ICD-9 chapter codes, (ii) top 50 ICD-9 codes, (iii) top 100 ICD-9 codes	(i) Micro F1 score of 0.76, (ii) Micro F1 score of 0.57, (iii) Micro F1 score of 0.51
Gangavarapu et al. [43]	Nursing notes	Deep learning	19 distinct ICD-9 chapter codes	Accuracy of 0.833
Samonte et al. [44]	Discharge summary	Deep learning	10 distinct ICD-9 codes	Precision of 0.780, Recall of 0.620, F1 score of 0.678
Obeid et al. [45]	Clinical notes	Deep learning	ICD-9 code from E950-E959	Area under the ROC curve score of 0.882, F-score of 0.769
Hsu et al. [46]	Subjective component	Deep learning	(i) 17 distinct ICD-9 chapter codes, (ii) 2017 distinct ICD-9 codes	(i) Accuracy of 0.580, (ii) Accuracy of 0.409
Xie et al. [47]	Diagnosis description	Deep learning	2833 ICD-9 codes	Sensitivity score of 0.29, Specificity score of 0.33
Singaravelan et al. [48]	Subjective component	Deep learning	1871 ICD-9 codes	Recall score for chapter code, 0.57; Recall score for block, 0.49; Recall score for three-digit code, 0.43; Recall score for full code, 0.45
Zeng et al. [49]	Discharge summary	Deep learning	6984 ICD-9 codes	F1 score of 0.42
Huang et al. [50]	Discharge summary	Deep learning	(i) 10 ICD-9 codes, (ii) 10 blocks	(i) F1 score of 0.69, (ii) F1 score of 0.72
Our study	Clinical notes	Deep learning	1131 ICD-10 codes	Precision of 0.96, Recall of 0.99, F-score of 0.98

## Data Availability

Not applicable.
